# The Exercise aNd hEArt Transplant (ENEA) Trial: A Randomized Controlled Trial of Cardiac Rehabilitation After Heart Transplantation

**DOI:** 10.3390/jcm15051832

**Published:** 2026-02-27

**Authors:** Paolo Pedersini, Alessandro Villaschi, Anastasia Toccafondi, Laura Antolini, Paola Grati, Ignazio Cusmano, Luca Mapelli, Matteo Gonella, Silvia Di Lauro, Riccardo Gonella, Gabriella Masciocco, Andrea Garascia, Nuccia Morici

**Affiliations:** 1 IRCCS Fondazione Don Carlo Gnocchi, Milan, Italy; 2 Department of Clinical and Experimental Sciences, University of Brescia, Brescia, Italy; 3 Department of Biomedical Sciences, Humanitas University, Pieve Emanuele, Milan, Italy; 4 De Gasperis Cardio Center, ASST Grande Ospedale Metropolitano Niguarda, Milan, Italy

**Keywords:** heart transplant, rehabilitation, exercise, telerehabilitation, physiotherapy

## Abstract

**Background**: Heart transplantation (HTx) remains the gold-standard therapy for patients with end-stage heart failure. Cardiac rehabilitation (CR) is a multidisciplinary intervention that improves cardiovascular prognosis and quality of life. The aim of this randomized controlled trial was to evaluate the impact of cardiac telerehabilitation on cardiovascular events after HTx. **Methods**: Forty patients who had undergone HTx were recruited at a single Italian institution and randomly allocated 1:1 to an experimental group (on-site CR followed by 12 weeks of telerehabilitation) or a control group (on-site CR followed by standard homecare and an exercise program). The primary outcome was a 6-month composite of major cardiovascular events, including acute allograft rejection, heart failure hospitalization, coronary allograft vasculopathy, stroke, and all-cause mortality. Secondary outcomes included return to work within 6 months, physical and functional activity levels and treatment adherence. **Results**: Forty patients were equally allocated to control and experimental groups, with well-balanced baseline demographic, clinical, and functional characteristics. At 6 months, the primary composite endpoint occurred in 35% of patients in both groups, with no significant between-group differences. Return to work was observed in 72.2% of the controls and 64.3% of intervention patients. Physical activity levels were comparable between groups, with most patients classified as sufficiently active. Adherence to the cardiac telerehabilitation program was complete in only 50% of the patients. **Conclusions**: In this randomized trial on HTx patients, a hybrid telerehabilitation program was as safe as standard care regarding major cardiovascular events at 6 months. The low adherence observed suggests that future digital interventions must focus on enhancing patient engagement.

## 1. Introduction

Heart transplant (HTx) is the gold standard for the treatment of selected patients with end-stage heart failure (HF). Despite significant advancements in transplant techniques, immunosuppressive therapies, and postoperative care, patients still experience, along with cerebral hypoperfusion, physical deconditioning and cognitive impairments related to inactivity in the pre-operative period that may impact optimal long-term outcomes [1].

Cardiac rehabilitation (CR) has been shown to improve functional capacity and reduce re-hospitalization in patients with HF [2]. Similarly, it may also play a relevant role in the comprehensive care and recovery of HTx patients, offering significant benefits to exercise capacity and reducing 1-year readmission rates [3]. Via CR programs tailored specifically for HTx patients, optimization of medication management, promotion of healthy lifestyle behaviours, and enhancement of overall well-being may also be achieved [4,5]. However, CR remains underutilized after HTx, with <50% of patients referred to it [6].

Early complications of HTx include hyperacute, cellular, and antibody-mediated rejection, right ventricular dysfunction, and infections related to immunosuppressive therapy. In particular, cytomegalovirus (CMV) infection represents one of the most relevant opportunistic pathogens in the post-transplant period. Hyperacute rejection, although rare due to current cross-matching strategies, can be rapidly fatal if not promptly recognized and treated [7]. Right ventricular dysfunction is a major cause of early morbidity and mortality, with severe right ventricular failure predicting poor outcomes and often requiring mechanical circulatory support [8,9,10]. In the long-term, cardiac allograft vasculopathy (CAV) emerges as the most critical complication, with increasing prevalence during the first 5–10 years after HTx and with a major impact on survival [11]. Moreover, CMV infection has been strongly implicated in the acceleration of CAV and graft dysfunction, even in the absence of overt clinical manifestations [9,12].

Telerehabilitation could play a crucial role in addressing the gaps in CR described above, as it may overcome relevant barriers, providing clinical benefits that are comparable to other strategies [13]. HTx recipients often face limitations in attending traditional center-based CR programs due to factors such as geographical distance, transport issues, and the compromised immune system of patients [14]. Telerehabilitation, which comprises both physical exercise and psychological support, provides viable solutions to these problems by utilizing remote monitoring, virtual communication, and digital health technologies to deliver rehabilitation services directly to patients’ homes [15]. This approach also allows for personalized exercise programs and real-time feedback and promotes convenience, accessibility, and continuity of care, as well as patient empowerment and self-management. Moreover, telerehabilitation reduces the risk of exposure to infections and facilitates the early detection of potential complications through continuous monitoring, ultimately improving patient outcomes and quality of life.

Therefore, we designed a randomized controlled trial (RCT) to evaluate the impact of cardiac telerehabilitation on cardiovascular events after HTx.

## 2. Materials and Methods

*Design and setting*

The design of the Exercise aNd hEArt transplant (ENEA) trial has been previously described [16]. Briefly, it is an RCT with two parallel arms and operates as a sub-study of the ongoing PROspective MulticEnter Registry in Patients with AcuTely DEcompensated Heart Failure Admitted to Cardiac RehabilitatiOn (PROMETEO). This RCT is conducted at the cardiac rehabilitation department of the IRCCS S. Maria Nascente, Milan, Italy.

*Eligibility criteria*

Patients aged ≥ 18 years old who have had HTx were considered eligible for inclusion. Exclusion criteria included inability to provide informed consent, inability to participate in face-to-face intervention sessions, contraindication to physical exercise, significant impairments in hearing, vision or communication, and need for regular treatment (such as chemotherapy or hemodialysis) that would interfere with the proper delivery of the intervention.

*Randomization and blinding*

Following baseline assessment, the participants were randomly assigned (1:1) to either an intervention or control group. Randomization were carried out using a routine based on pseudo-random numbers generated by Statistical Analysis Software (SAS) package version 9.4. Briefly, a sequence of 40 random numbers following the normal distribution was generated using a specific seed code. The sequence of numbers was then sorted so that the first 20 subjects were allocated to the experimental arm and the second 20 subjects to the control arm. Treatment groups were identified with a color code so that allocation was blinded. Due to the nature of the intervention, blinding of participants and clinicians delivering the intervention was not possible. Outcome assessors were blinded to group allocation.

*Data collection*

Participants were evaluated at baseline (T0: before the beginning of the intervention program), post-CR (T1: after facility-based CR), and at follow-up, 6 months after the beginning of the intervention (T2).

*Experimental group*

In the “experimental” group, patients participated in the standard daily rehabilitation program for heart transplant (HTx) patients undergoing cardiac rehabilitation (CR), followed by a prescribed telerehabilitation phase. During the facility-based phase, patients performed 90–120 min of physical activity per day, divided into multiple sessions based on their clinical status and exercise tolerance. Sessions initially consisted of short, frequent bouts of exercise (3–4 times in the morning and afternoon), progressively consolidated into longer sessions combining endurance and resistance training. Endurance work was performed with a treadmill, exercise bike, or walk, and was gradually increased from low-intensity interval training to continuous training, with intensity adjusted based on subjective fatigue [Borg Rate of Perceived Exertion (RPE) 13/20 or Borg Category-Ratio 10 (CR10) 4/10]. When feasible, a cardiopulmonary exercise test (CPET) was performed, and exercise intensity was maintained in the range of 70–85% of peak oxygen uptake (VO2 peak). Resistance work consisted of lower limb exercises and, when appropriate, upper limb exercises, with 3–4 sets of 10 repetitions per muscle group. Intensity was titrated using the OMNI-Resistance Exercise Scale (OMNI-RES) (target level 4). Following discharge, patients transitioned to a 12-week home-based telerehabilitation program, which was interrupted if hospitalization occurred. A specialist cardiologist defined the individualized plan, implemented by a multidisciplinary team via a web platform. Patients accessed asynchronous content daily and participated in scheduled synchronous sessions (twice weekly for 4 weeks, then weekly for 8 weeks). The program aimed to maintain 50–60 min/day alternating between endurance and resistance exercise, adjusting the demands according to each patient’s condition and individual progress. Training was supported by vital sign monitoring (heart rate, blood pressure, peripheral oxygen saturation, weight), physiotherapist supervision, and psychological support. The physician contributed as necessary to synchronous sessions and oversaw long-term clinical stability.

*Control group*

In the “control” group, patients received the same facility-based rehabilitation treatment as those in the experimental group. At discharge, they received a prescription for home exercises and guidelines for maintaining healthy behaviours over an additional 12-week period, in accordance with standard clinical practice. The 5-days per week program included upper and lower limb strength training and endurance exercises. Effort intensity was adjusted according to subjective perceptions of fatigue (Borg RPE 13/20 or Borg CR10 4/10) and/or heart rate, maintained within 70–85% of VO2 peak for endurance training. Resistance exercises were performed using natural or external loads, with a customized number of sets, repetitions, and overload, aiming to reach a perceived muscle fatigue level of 4 on the OMNI-RES.

*Clinical outcomes*

The primary clinical outcome was a composite of major cardiovascular events at 6 months, including acute allograft rejection, hospitalization for HF, CAV, stroke, and all-cause mortality. Secondary outcomes included return to work within 6 months after transplantation, the level of physical and functional activity achieved at the end of the rehabilitation program, and adherence to the prescribed treatment. Physical activity was assessed using the International Physical Activity Questionnaire (IPAQ) [17], which classifies patients as inactive, moderately active, or active. In the experimental group, treatment adherence was evaluated for both the telerehabilitation component and the psychological support program. To this end, a dedicated analysis of rehabilitation adherence was performed to assess patient participation in scheduled sessions and to examine the impact of adherence on medium-term clinical and functional outcomes.

*Statistical analysis*

Continuous variables are reported as median and interquartile range or mean ± standard deviation, as appropriate, while categorical variables are presented as absolute numbers and percentages. Baseline characteristics were compared between the control and experimental groups using the Student’s *t* test or the Mann–Whitney U test for continuous variables, depending on data distribution, and the χ^2^ test or Fisher’s exact test for categorical variables. The primary endpoint, defined as the composite of cardiovascular events at 6 months, was compared between groups using the χ^2^ test. Secondary endpoints, including return to work and physical activity categories derived from the IPAQ, were analyzed descriptively and compared between groups using χ^2^ or Fisher’s exact tests, as appropriate. Adherence analyses were conducted exclusively within the experimental group. Patients were categorized according to their level of adherence to the telerehabilitation and psychological support program (non-adherent, telerehabilitation only, or full intervention). Event rates were calculated for each adherence category and compared descriptively due to the limited sample size. A two-sided *p*-value < 0.05 was considered statistically significant. Statistical analyses were performed using STATA.

## 3. Results

### 3.1. Demographic and Clinical Characteristics of the Patients at Baseline

A total of 40 patients were enrolled, with 20 assigned to the control group and 20 to the experimental group. Baseline demographic and clinical characteristics are reported in Table 1. The two groups were well-balanced. Female sex was present in 37.5% of the overall cohort (45% in the control group and 30% in the experimental group). Median age was similar in both groups (51 years vs. 52 years). Median time from HTx to beginning CR was 43 days for the overall cohort. Active smoking was more prevalent in the control group (78.9% vs. 35%), whereas a greater proportion of former smokers was observed in the experimental group (50% vs. 21.1%). The prevalence of dyslipidemia, hypertension, and other comorbidities was comparable between groups.

Clinical and laboratory parameters, including renal function, echocardiographic measures, and biomarkers, were broadly similar across groups. Total and low-density lipoprotein (LDL) cholesterol levels were higher in the experimental group, whereas body mass index and other biochemical variables were comparable (Table 1). Pharmacological therapy was evenly distributed, with virtually all patients being treated with a combination of corticosteroid, calcineurin-inhibitor (cyclosporine or tacrolimus), and mycophenolate mofetil/mycophenolic acid. Functional assessments revealed slightly greater walking test performance in the control group (Table 1).

### 3.2. Primary Endpoint

The primary clinical outcome, a composite of relevant cardiovascular events at 6 months, including acute allograft rejection, hospitalization for heart failure, CAV, stroke, and all-cause mortality, showed no statistically significant difference between the control and experimental groups. In both the control group (*n* = 20) and the experimental group (*n* = 20), 65.0% experienced an absence of the composite event, while 35.0% experienced the presence of the event. No statistically significant differences were observed between the two groups (Table 2).

### 3.3. Secondary Endpoint

Among the employed patients, return to work within 6 months after transplantation was observed in 13 out of 18 subjects (72.2%) in the control group and in 9 out of 14 subjects (64.3%) in the experimental group, while 5 patients in each group did not resume employment during the follow-up period. Physical activity levels, assessed using the IPAQ, were available for 19 patients in each group. At the end of the rehabilitation program, the majority of patients in both groups were classified as sufficiently active [11/19 (57.9%) in the control group and 10/19 (52.6%) in the experimental group]. A smaller proportion of patients were classified as active [3/19 (15.8%) in the control group and 2/19 (10.5%) in the experimental group], whereas 5/19 (26.3%) in the control group and 7/19 (36.8%) in the experimental group were classified as inactive (Table 3).

### 3.4. Adherence to Telerehabilitation

This analysis focused exclusively on the experimental group and evaluated the relationship between adherence to the telerehabilitation and psychological support via telemedicine and the occurrence of events. In the experimental group (*n* = 20), adherence to the combined telerehabilitation and psychological support program was heterogeneous. Ten patients (50.0%) were non-adherent, having not participated in any scheduled intervention sessions. Two patients (10.0%) adhered only to the telerehabilitation component, while eight patients (40.0%) completed the full intervention, including both telerehabilitation and psychological support. Overall, 7 clinical events were recorded among the 20 patients, corresponding to an overall event rate of 35.0%. When stratified by adherence level, the event rate was 30.0% (3/10) in non-adherent patients. Patients who received only telerehabilitation showed an event rate of 50.0% (1/2), while those who completed the entire intervention exhibited an event rate of 37.5% (3/8) (Table 4).

## 4. Discussion

The ENEA trial provides critical insights into the feasibility and safety of cardiac telerehabilitation for HTx patients, a population traditionally managed with high-intensity, center-based supervision. Although a small cohort, this is the first RCT reported in the literature featuring early (median time from HTx to CR 43 days) stationary secondary rehabilitation and a tertiary rehabilitation approach. Our findings suggest that a hybrid model, combining initial facility-based rehabilitation with a subsequent 12-week remote phase, is as clinically safe as the traditional standard of care. This safety profile is paramount, given the complexity of the post-transplant period and the inherent risks of immunosuppression and graft rejection. While our study was not designed to capture granular changes in functional outcomes, the observed low adherence in the experimental group highlights a substantial challenge. To unlock the full potential of digital health in this context, future trials must not only implement strategies to improve patient engagement but also incorporate more comprehensive and sensitive measures of functional capacity.

The importance of cardiac CR in the post-transplant period cannot be overstated. HTx patients often suffer from significant physical deconditioning, muscle atrophy, and reduced aerobic capacity due to pre-operative heart failure and the effects of surgery and immunosuppression. Systematic reviews and meta-analyses, such as the 2017 Cochrane review by Anderson et al., have consistently shown that exercise-based CR significantly improves exercise capacity and quality of life in this population [3]. Despite these benefits, CR remains underutilized, with fewer than 50% of eligible HTx patients being referred to such programs [6]. Our results align with the broader evidence supporting the safety of home-based cardiac rehabilitation models [18]. While these studies typically compare home versus center-based care, our trial builds upon this foundation by showing that a digitally supervised home program is as safe as a traditional, non-supervised home exercise prescription in the HTx patients. However, the specific context of HTx introduces unique variables. Unlike general HF populations, where telerehabilitation has shown high success rates, HTx patients face the constant threat of opportunistic infections, such as CMV, which has been implicated in the acceleration of CAV. In this regard, cardiac telerehabilitation offers a theoretical advantage by reducing hospital exposure and the associated risk of nosocomial infections. However, this benefit can only be realized if patients remain engaged with the program. The primary endpoint of this trial was a composite of major cardiovascular events designed to assess the clinical impact of our hybrid telerehabilitation model. We observed no significant difference in the occurrence of these events between the intervention and control groups. Our findings suggest that with appropriate protocols, remote management does not increase the risk of adverse clinical outcomes. However, the lack of an evident benefit in the experimental arm strongly suggests that the program, as implemented, was insufficient to change the clinical trajectory of our patients. This could be a direct consequence of the suboptimal adherence observed. Therefore, our primary finding should be interpreted not as a failure of the telerehabilitation concept itself, but as a reflection of the challenges in its practical application. The primary explanation for the comparable results between groups in our study, rather than the hypothesized superiority of cardiac telerehabilitation, lies in the low adherence to the remote program. Adherence to digital health interventions is a complex phenomenon, and in the case of heart transplant recipients, it is likely hindered by a combination of medical fatigue and psychological burden. Patients who have recently undergone a lifesaving but traumatic surgical procedure often experience a period of “medical overload”, where the management of complex immunosuppressant regimens and frequent clinical visits takes precedence over elective exercise programs [14]. Furthermore, the transition from the highly supportive and supervised environment of a rehabilitation facility to the relative isolation of a home-based program can lead to a loss of motivation. The “digital divide” also remains a relevant factor; despite the increasing presence of technology, technical difficulties with web platforms and a lack of digital literacy can frustrate patients, leading to early dropout [19]. The unexpectedly high rate of non-adherence observed in our experimental group deserves further consideration. While previous experiences in heart failure have reported a good feasibility of telerehabilitation programs, adherence in the early post-transplant phase may be influenced by specific barriers [20]. Recent qualitative evidence suggests that kinesiophobia, perceived physical limitations, and psychological distress represent significant obstacles to physical activity in heart transplant recipients [21]. In addition, this period still corresponds to a phase of clinical fragility and high medical burden, which may reduce the patients’ readiness to engage in structured remote programs. Furthermore, technological demands and digital literacy may have acted as additional barriers. These findings highlight that adherence in this population is likely multifactorial and emphasize the need for future trials to incorporate a structured assessment of psychological, clinical, and technological determinants of engagement.

Beyond hard clinical endpoints, a key objective of rehabilitation is the restoration of a patient’s functional and social life, with return to work and physical activity levels being crucial indicators of success. In our trial, we observed that a majority of previously employed patients in both groups successfully returned to work. Return to work is a complex outcome influenced by psychosocial factors, not just physical capacity [22]. Regarding physical activity, our findings show that at the end of the program, the majority of participants in both arms were classified as “sufficiently active”. This is a positive outcome, indicating that both rehabilitation models were effective in guiding patients towards the recommended activity levels. However, it is noteworthy that a substantial proportion of patients remained “inactive”, and the telerehabilitation arm did not demonstrate superiority in promoting higher activity levels. This result aligns with the broader literature suggesting that while supervised exercise effectively improves functional capacity [23], translating these gains into sustained, self-directed physical activity is a major challenge. Simply providing a remote tool may not be enough to overcome barriers like low self-efficacy or fear of exertion. Future interventions must incorporate more robust behavioral change techniques to foster long-term self-management and ensure that the benefits of rehabilitation extend beyond the structured program phase [24].

### Study Limitations

This study has several important limitations. Firstly, and most significantly, the trial was conducted at a single center with a small sample size. While sufficient for a preliminary assessment of safety and feasibility, the study was not statistically powered to detect significant differences in clinical endpoints, including the primary composite outcome. Consequently, our findings regarding the non-inferiority of telerehabilitation should be considered hypothesis-generating rather than definitive. The small subgroups in the adherence analysis also prevent any meaningful conclusions about the relationship between patient engagement and clinical outcomes.

Secondly, the high rate of non-adherence (50%) in the experimental group represents a major limitation. This not only weakened the potential therapeutic effect of the intervention, making it difficult to assess its true efficacy, but also raises questions about the acceptability and real-world applicability of this specific telerehabilitation model. Thirdly, our assessment of functional outcomes had its limitations. While we collected data on return to work and used the IPAQ for physical activity, we did not incorporate more objective or granular measures. This limited our ability to estimate clinical changes and potential differences in functional recovery and behavioral change between the groups. In addition, some secondary outcomes initially defined as related to functional capacity were not reported because they were not collected for all patients. Finally, the follow-up period was limited to 6 months. This timeframe is adequate for assessing short-term safety and recovery but is insufficient to evaluate the long-term impact of rehabilitation on outcomes such as the progression of CAV, chronic quality of life, or survival. Future larger, multi-center trials with longer follow-up and more robust measures of adherence and functional status are warranted to overcome these limitations and definitively establish the role of telerehabilitation in HTx care.

## 5. Conclusions

In this RCT, a hybrid rehabilitation program including telerehabilitation showed no significant difference in the rate of major cardiovascular events at 6 months compared with standard care, thereby establishing its clinical safety in the heart transplant population. However, the intervention did not demonstrate superiority in improving return-to-work rates or physical activity levels, a finding likely influenced by the low adherence to the remote program. These results suggest that while telerehabilitation is a safe option, future models must integrate more effective strategies to enhance patient adherence and engagement. Future research should therefore focus on developing digital interventions that are not only technologically advanced, but also psychologically supportive and personalized, to maximize long-term functional benefits.

## Figures and Tables

**Table 1:** Demographic and clinical characteristics of the patients at baseline and pharmacological therapy and functional assessment.

	Overall (*n* = 40)	Control Group (*n* = 20)	Experimental Group (*n* = 20)
**Characteristic, *n* (%)**			
Female	15 (37.5)	9 (45)	6 (30)
Median age [range], yrs	51 [44.5–58.25]	51 [38–56]	52 [47.25–60]
Smoker *	22 (55)	15 (78.9)	7 (35)
Ex-smoker	14 (35)	4 (21.1)	10 (50)
**Medical history, *n* (%)**			
Ischemic etiology	29 (72.5)	16 (80)	13 (65)
Dyslipidemia	28(70)	15 (75)	13 (65)
Hypertension	8 (20)	2 (10)	6 (30)
Polyneuropathy	17 (42.5)	9 (45)	8 (40)
BPCO	3 (7.5)	2 (10)	1 (5)
Diabetes	3 (7.5)	1 (5)	2 (10)
Myelopathy	2 (5)	2 (10)	0 (100)
**Clinical parameters, median [IQR]**			
Days before admission to CR	43 [27.5–68.5]	43 [27–101]	44 [28–68]
Pericardial effusion, *n* (%)	11 (28.2)	5 (25)	6 (31.6)
Body mass index kg/m^2^	22.5 [20.2–25.6]	23.1 [21.3–25.6]	20.8 [20.0–25.6]
Cholesterol, total *	218.0 [180.0–270.0]	192.0 [171.0–218.0]	250.5 [197.2–305.2]
Cholesterol, LDL *	107.0 [94.0–146.5]	100.0 [85.0–123.0]	129.5 [102.5–172.2]
Triglycerides	153 [119–275]	169 [126–248]	146 [116–282]
VTDi	50.9 [41.3–58.7]	46 [41–53]	56 [44–64]
IVS	10 [10–11]	10 [10–11]	10 [10–11]
PP	10 [9–11]	10 [9–11]	10 [9.2–10.8]
IVRT	81.5 [67–97.5]	84 [69.8–95.5]	76.5 [67.2–99.8]
MR I, *n* (%)	19 (45)	5 (25)	14 (70)
MR II, *n* (%)	20 (50)	14 (70)	6 (30)
MR III, *n* (%) *	1 (5)	1 (5)	0 (0)
HB	9.9 [9.4–10.4]	9.9 [9.4–10.4]	9.9 [9.6–10.4]
eGFR	48.8 [32.7–91.0]	48.8 [34.6–91]	48.2 [30.1–82.3]
SIV	10 [10–11]	10 [10–11]	10 [10–11]
FE	59.5 [57–61]	60.0 [57.8–62.2]	59.0 [57.0–60.0]
E/A	1.8 [1.6–2.1]	1.7 [1.6–1.9]	1.9 [1.8–2.3]
E/E	7.5 [6.6–9.5]	7.5 [6.7–8.3]	8.0 [6.5–10.1]
Nt Probnp	4743 [2464–9481]	4743 [3128–7111]	4740 [2281–10405]
Potassium	4.3 [4.1–4.7]	4.2 [4.1–4.7]	4.3 [4.2–4.6]
Sodium	138 [137–139]	138.0 [137.0–138.2]	139.0 [137.0–139.0]
Urea	61 [52.2–85.3]	59.5 [52.2–86.0]	62.0 [50.5–80.8]
MCV	90.9 [89.2–93]	91.2 [88.1–94.7]	90.8 [90.2–92.2]
WBC	7.2 [5.1–8.6]	6.97 [5.82–8.02]	7.64 [4.82–8.72]
AST	21.0 [16.3–28.0]	23.0 [17.0–26.5]	20.1 [16.0–28.8]
ALT	25.8 [15.5–43]	26.0 [16.7–43.0]	24.0 [14.8–42.2]
Bilirubin *	0.7 [0.5–0.8]	0.8 [0.6–1.0]	0.6 [0.4–0.8]
BEM Num	3 [2–4]	4 [2–4]	4 [2.5–4]
**Pharmacological therapy, *n* (%)**			
Cortisone	40 (100)	20 (100)	20 (100)
Tacrolimus	20 (50)	12 (60)	8 (40)
Ciclosporin	19 (47.5)	7 (35)	12 (60)
Mycophenolate mofetil/mycophenolic acid	38 (95)	19 (95)	19 (95)
Beta blockers	7 (17.5)	5 (25)	2 (10)
ACE inhibitor	2 (5)	2 (10)	0 (0)
Statin	8 (20)	5 (25)	3 (15)
Antivirals	28 (70)	14 (70)	14 (70)
Antibiotics	30 (75)	17 (85)	13 (65)
**Functional assessment, median [IQR]**			
WT dist (m)	137 [0–289]	160 [0–324.75]	132 [0–234.25]
WT perc	12.1 [0–24.3]	14.7 [2.0–26.4]	10.5 [0–20.6]
WT borg	4 [3–5.5]	3.5 [3–6.25]	4 [3–5]

(*) indicates statistically significant differences between groups (*p* < 0.05). Abbreviations: yrs: years; CR: cardiac rehabilitation; IQR: interquartile range; VTDi: left ventricular end-diastolic volume index; IVS: interventricular septum thickness; PP: posterior wall thickness; IVRT: isovolumic relaxation time; MR I–III: mitral regurgitation grades I–III; HB (Hb): hemoglobin; eGFR: estimated glomerular filtration rate; SIV: interventricular septum (septal thickness measurement); FE (EF): ejection fraction; E/A: ratio of early (E) to late (A) diastolic transmitral flow velocities; E/E′ (E/E): ratio of early transmitral flow velocity to early diastolic mitral annular velocity; NT-proBNP (Nt Probnp): N-terminal pro-B-type natriuretic peptide; MCV: mean corpuscular volume; WBC: white blood cell count; AST: aspartate aminotransferase; ALT: alanine aminotransferase; BEM Num: number of endomyocardial biopsies. WT dist: walking test distance; WT perc: walking test percentage of predicted value; WT borg: Borg rating of perceived exertion scale.

**Table 2:** Primary endpoints: rate of composite of relevant cardiovascular events.

Primary Endpoint	Control (*n* = 20)	Experimental (*n* = 20)
No event rate, % (*n*)	65.0% (13)	65.0% (13)
Event rate, % (*n*)	35.0% (7)	35.0% (7)

**Table 3:** Secondary endpoints (return to work and physical activity).

Secondary Endpoint	Category	Control Group, *n* (%)	Experimental Group, *n* (%)
Return to work	Yes	13 (72.2)	9 (64.3)
	No	5 (27.8)	5 (35.7)
Employed subjects, *N*	Total	18	14
Physical activity (IPAQ score)	Active	3 (15.8)	2 (10.5)
	Sufficiently active	11 (57.9)	10 (52.6)
	Inactive	5 (26.3)	7 (36.8)
Available subjects, *N*	Total	19	19

Abbreviation: IPAQ: International Physical Activity Questionnaire.

**Table 4:** Adherence of telerehabilitation and psychological support for the experimental group.

Adherence Level	Frequency (No. of Patients)	Percentage, %	Events, *n*/N	Event Rate, %
Non-adherent (no intervention)	10	50.0	3/10	30.0
Only telerehabilitation	2	10.0	1/2	50.0
Completed	8	40.0	3/8	37.5
Total	20	100.0	7/20	35.0

## Data Availability

The data presented in this study are available on reasonable request.
